# Avian Haemosporidians Infecting Short- and Long-Distance Migratory Old World Flycatcher Species and the Variation in Parasitaemia After Endurance Flights

**DOI:** 10.1007/s11686-023-00710-0

**Published:** 2023-08-17

**Authors:** Tamara Emmenegger, Sara Riello, Raffaella Schmid, Lorenzo Serra, Fernando Spina, Steffen Hahn

**Affiliations:** 1 Bird Migration department, Swiss Ornithological Institute, Sempach, Switzerland; 2 Molecular Ecology and Evolution Lab, Lund University, Lund, Sweden; 3 Riserva Naturale Statale “I Sole di Ventotene e S.Stefano”, Ventotene, Italy; 4 Area Avifauna Migratrice, Istituto Superiore per la Protezione e la Ricerca Ambientale (ISPRA), Ozzano Emilia, , BO Italy

**Keywords:** *Haemoproteus*, Muscicapidae, Migration strategy, Parasitaemia, *Plasmodium*

## Abstract

**Purpose:**

Avian haemosporidians are widespread parasites, occurring in many bird families and causing pathologies ranging from rather benign infections to highly virulent diseases. The state of knowledge about lineage-specific intensities of haemosporidian infection (i.e., parasitaemia) is mainly based on infection experiments conducted under laboratory conditions. The levels and range of parasitaemia in natural host–parasite associations as well as their influencing factor remain largely unexplored.

**Methods:**

Thus, we explored the parasitaemia of four songbird species (i.e., European Robins, Black and Common Redstarts and Whinchats) during migration by screening individuals upon landing on an insular passage site after extensive endurance flights to (1) describe their natural host–parasite associations, (2) quantify parasitaemia and (3) explore potential host- and parasite-related factors influencing parasitaemia.

**Results:**

We found 68% of Whinchats to be infected with haemosporidians, which is more frequent than any other of the studied host species (30–34%). Furthermore, we confirmed that parasitaemia of *Haemoproteus* infections was higher than average *Plasmodium* infections. Median parasitaemia levels were rather low (parasite cells in 0.01% of hosts’ red blood cells) and varied largely among the different parasite lineages. However, we found four individuals hosting infections with parasitaemia higher than typical chronic infections.

**Conclusions:**

Based on the known transmission areas of the respective lineages, we argue that these higher intensity infections might be relapses of consisting infections rather than acute phases of recent primary infections.

## Introduction

Migratory insectivorous bird species track suitable conditions for arthropods year-round. As haemosporidian blood parasites are transmitted by ornithophilic insects anywhere along migration routes, migrants often harbour a more diverse spectrum of parasites than resident hosts [1]. Particularly haemosporidians of the genera *Haemoproteus* and *Plasmodium* are cosmopolitan, transmitted by Hippoboscidae, Ceratopogonidae and Culicidae vectors, respectively. These vector are ectotherms, and their development, abundance and activity highly depends on ambient temperature [2, 3]. Therefore, the probability of transmission may vary among the biomes visited by migratory birds throughout their annual cycle.

Infections can cause symptoms ranging from mild fever to severe anaemia entailing reduced mobility and lethargy or even death [4]. After the initial acute infection phase with high number of infected host erythrocytes, the number of parasites usually drops to low chronic levels. This chronic infection phase is often sustained life-long, can rarely be fully cleared, and host individuals can undergo seasonal or stress-related relapses, during which the parasitaemia and symptoms can temporarily reach levels typical of primary infections again. Seasonal relapses are assumed to occur during challenging annual-cycle periods of the host, like, e.g., migration, since a surplus of energy is allocated to preparing and accomplishing migratory flights paralleled by reduced immune defence [5–7].

This raises the question, if migrating birds which are infected with haemosporidian parasites can show high parasitaemia (higher than expected for typical chronic infections), especially if they recently accomplished endurance flights during their seasonal journey.

So far, data about host species- and parasite lineage-specific parasitaemia ranges are scarce as many studies on avian haemosporidians solely use molecular methods. Here, we combined molecular detection methods with quantification by classical microscopy to assess the range of haemosporidian parasitaemia of passerines on their migratory flights. We sampled short- and long-distance migratory species shortly after crossing major ecological barriers on their pre-breeding migration to detect haemosporidian infections and assess parasitaemia. We briefly describe the host-specific prevalence and diversity of *Haemoproteus* and *Plasmodium* infections. We finally quantify parasitaemia and investigate if parasitaemia levels are more driven by parasite genus, host species or host migration strategy. Besides parasitaemia levels typical for chronic infections, we expect to find cases of high parasitaemia in hosts after endurance flights. If long-distance migratory species are more prone for relapses, as they perform longer or more endurance flights than short-distance migrants, we expect differences in average or maximal parasitaemia depending on the migration strategy of the hosts.

## Materials and Methods

### Study Site and Host Species

We investigated haemosporidian infections of birds captured during spring migration in 2016 and 2017 on Ventotene Island (40°47′51″N 13°25′48″E), the first stopover site after barrier crossing. We randomly selected individuals of four Muscicapidae species: European Robins (*Erithacus rubecula*; *n* = 63; 45 females, 16 males, 2 unidentified), Black Redstarts (*Phoenicurus ochruros*; *n* = 41; 29 females, 12 males), Common Redstarts (*P. phoenicurus*; *n* = 107, 49 females, 58 males) and Whinchats (*Saxicola rubetra*; *n* = 140; 52 females, 88 males). Robins and Black redstarts are short-distance and the latter two long-distance migrants. While all birds must have crossed the Mediterranean Sea (≈470 km), the long-distance migrants have also crossed the Sahara Desert before arriving on Ventotene. In another study of this collaboration project, we also investigated how infections and migration strategy (i.e., short- vs. long-distance migration) may relate to body condition, haemoglobin content and aerobic performance [8].

### Blood Sampling, Determination of Infection Status and Analysis of Prevalence of Hosts

We sampled blood by piercing the brachial vein with a hypodermic needle and collecting a drop of blood with a heparinized capillary. We prepared two blood smears per individual and stored the remaining blood in SET buffer (0.015M NaCl, 0.05M Tris-HCl, 0.001M EDTA, pH8).

To assess the infection status, we extracted DNA with spin-columns (DNeasy blood and tissue kit, Qiagen) and performed a nested PCR (according to [9]; primers: nested1 = HaemNF1/HaemNR3, nested2 = HaemF/HaemR2; thermal profile of first PCR is 3 min at 94 °C + [30s at 94 °C + 30s at 50 °C + 45 s at 72 °C] for 20 cycles + 10 min at 72 °C, and the one of the second PCR is identical but with 35 cycles instead of 20 cycles). Samples with unclear nested PCR results (weak bands) were rerun and, together with all positive samples, additionally screened by microscopy to reduce the risk of false negative results. We calculated total and parasite genus specific prevalence (= number of infected/tested individuals) and tested for differences in prevalence related to the hosts in a generalised linear model (function gLm, R package stats, formula: infection status [0,1]  ~ host species + host sex, family ‘binomial’).

### Sequencing and Analysis of Haemosporidian Parasite Lineages

To identify parasite lineages in the infected host individuals, the products of parasite-positive and unclear PCRs were purified, entered single-pass sequencing (both HaemF and HaemR2) and the resulting chromatograms/sequences had been checked/edited in BioEdit [10]. The chromatograms of two samples showed mixed template patterns to a degree that neither genus nor lineage could be assigned. The chromatograms of 125 PCR-positive samples were clean or with only single mixed bases, which did not hamper lineage assignment. The consensus sequences were blasted against lineages registered in the MalAvi database ([11], accessed on 18/03/2022), to detect known lineages (100%-match with a known lineage) and to describe new lineages (no full-match with any known lineage).

To compare lineage diversity and composition among host species, we first calculated relative parasite lineage richness by dividing the absolute number of lineages by the number of infected individuals. Then, we accounted for the detectability of rare lineages by calculating rarefied parasite lineage richness (function rarefy, R package vegan). To get insight into the host-specific lineage compositions, we plotted a Venn diagram with the number of unique and shared among lineages (function venn.diagram, R package VennDiagram) and determined the pairwise Chao dissimilarity indices for the lineages hosted by each species (function vegdist, R package vegan).

### Microscopy and Analysis of Parasitaemia in Individual Hosts

To determine parasitaemia (= number of infected/number of examined erythrocytes), we screened the blood smears of samples with parasite-positive and unclear PCR results by examining 100 microscopic fields with 1000 magnification with a light microscope (Primo Star, Carl Zeiss AG). The parasites were counted and the total number of inspected erythrocytes was extrapolated from five representative pictures (following [12]). In case we did not find parasite-infected erythrocytes (*n* = 18) or only extracellular stages of parasites (*n* = 2) in blood smears of PCR-positive samples with clear chromatograms, we set the parasitaemia to half of the minimum detectable parasitaemia [13]. If we did not find any parasite in samples with unclear PCR results and unclear chromatograms, we set the infection status to ‘uninfected’.

We log-transformed parasitaemia data for the statistical analyses (function logst, see [14]) to avoid zero-inflation. Finally, we statistically investigated potential sources of variation in parasitaemia by running two linear models (LMs): The first LM was designed to test for differences in parasitaemia related to parasite genus or host species as well as their interactions (function lm, R package stats, formula: parasitaemia ~ parasite genus * host species). The second LM was designed to test for differences in parasitaemia related to parasite lineage (each lineage compared to unidentified infections as the reference level), controlling for potential differences between host species (function lm, R package stats, formula: parasitaemia ~ parasite lineage + host species). All analyses were performed in R 4.1.2 [15].

## Results

### Haemosporidian Prevalence

**Fig. 1:**
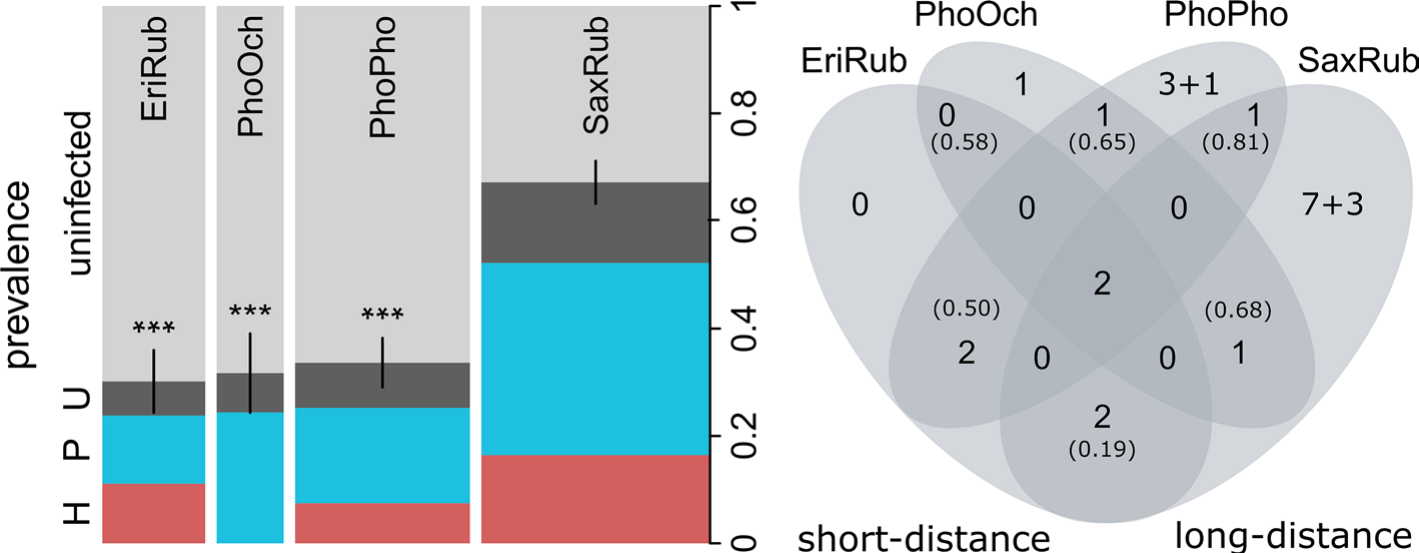
Haemosporidian parasitism in two short-distance migrant bird species (EriRub = European Robins, *n* = 63; PhoOch = Black Redstarts, *n* = 41) and two long-distance migrants (PhoPho = Common Redstarts, *n* = 107; SaxRub = Whinchats, *n* = 140) during pre-breeding migration. The left panel shows host-specific prevalence (proportion of infected individuals) with standard error for total prevalence (whiskers). Total prevalence comprises infections with *Haemoproteus* (H, red), *Plasmodium* (P, blue) and infections, where genera (H or P) remained unknown (U, dark grey). The widths of the bars represent sample sizes. Significant differences in prevalence between Whinchats (reference species) and the other host species are indicated with asterisks (*** = *p* < 0.001). The right panel shows the numbers of genetic lineages uniquely found in one or shared among the four host species. Newly described lineages are added to previously known lineages after the ‘ + ’ sign. Pairwise Chao distance indices are given in brackets, wherein low indices indicate high similarity in parasite lineage assemblage)

The haemosporidian prevalence in Whinchats 67.9% (SE =  ± 0.04) was by far the highest and, therefore, taken as reference level in the statistical models (Fig. 1, left panel). The prevalence of European Robins (30.2%, SE =  ± 0.06), Black Redstarts (31.7%, SE =  ± 0.07) and Common Redstarts (33.7%, SE =  ± 0.05) were statistically lower (GLM: slope_EriRub_ = − 1.58 [± 0.34], z = − 4.60, *p* < 0.001; slope_PhoOch_ = − 1.55 [± 0.39], z = − 3.96, *p* < 0.001; slope_PhoPho_ = − 1.41 [± 0.27], z = − 5.16, *p* < 0.001). Prevalence did not significantly differ between sexes of hosts (GLM: slope_m→f_ = 0.20 [± 0.24], z = 0.81, *p* = 0.42). Apart from the absence of *Haemoproteus* infections in Black Redstarts, the proportions of *Haemoproteus* (22–37%), *Plasmodium* (42–53%) and unknown infections (21–25%) were comparable in all host species (Fig. 1, left panel).

### Parasite Lineage Assemblage

**Fig. 2:**
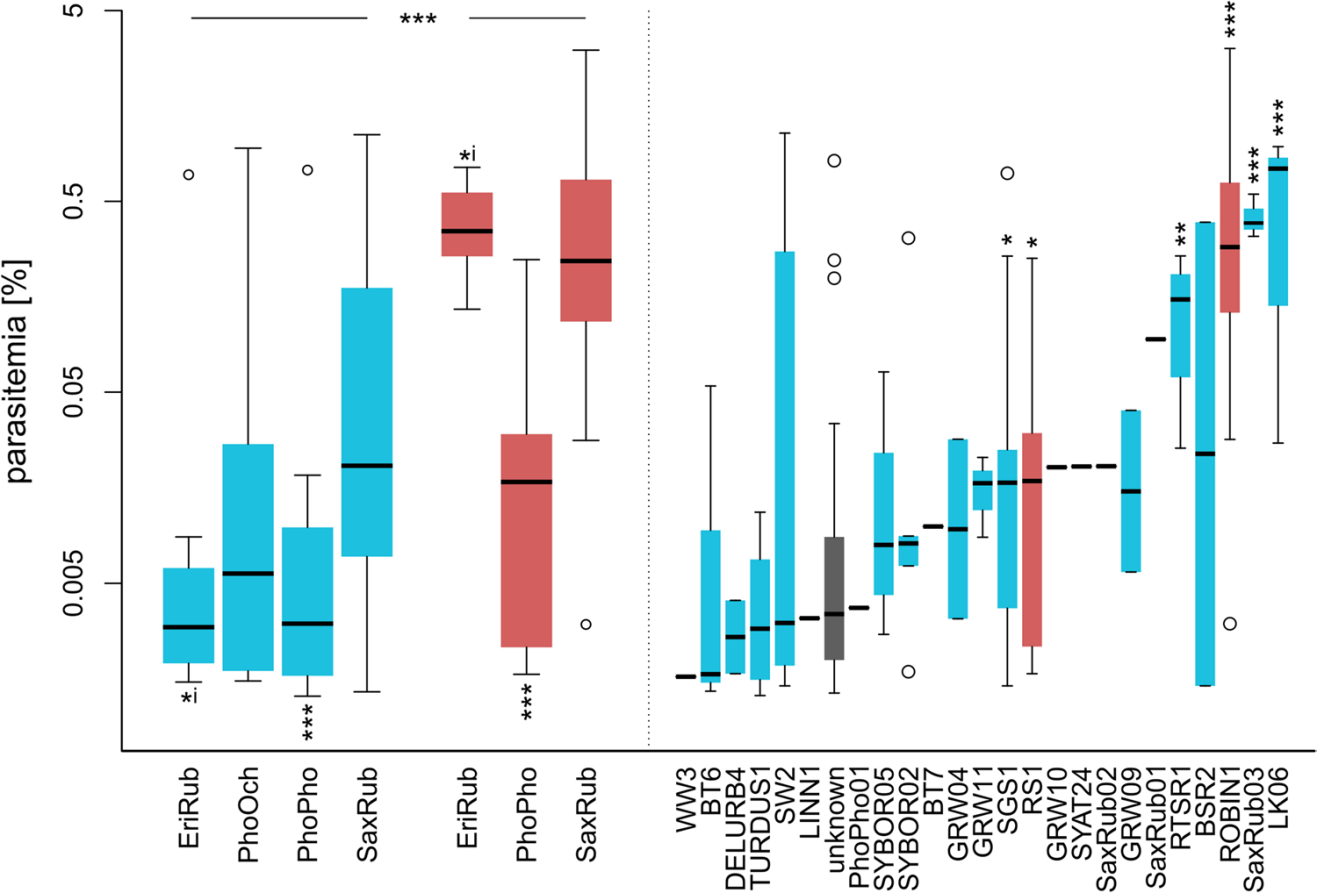
Haemosporidian parasitaemia of four migrant passerine host species during their pre-nuptial migration period (EriRub = European Robins, PhoOch = Black Redstarts, PhoPho = Common Redstarts, SaxRub = Whinchats). Parasite genera and parasite lineages are symbolized with *Plasmodium* in blue, and *Haemoproteus* in red. The left panel shows host species- and parasite genus-specific parasitaemia (median and quartiles). Outliers (four quartile ranges) are given as circles. The right panel shows lineage-specific parasitaemia (lineages sorted by median parasitaemia). Both panels: statistical significance is shown as *** (*p* < 0.001), ** (*p* < 0.01), * (*p* < 0.05). Asterisks above and below the boxes indicate significantly higher and lower parasitaemia, respectively. An ‘i’ symbolise significant interaction between parasite genus and host species

We found 24 genetically unique haemosporidian lineages lineages (see Fig. 2, right panel) in 125 infected hosts, wherein two *Haemoproteus* and 18 *Plasmodium* lineages had been known before (i.e., recorded in the MalAvi database); four *Plasmodium* lineages had not been described yet (P-PhoPho01 and P-SaxRub01-03; Genebank accession numbers: ON931619-22).

Most lineages (63%) were unique to one host species, seven lineages were found in two out of the four host species and two lineages were found in all four host species (Fig. 1, right panel). We found relative lineage diversities of 0.40 lineages per infected European Robin, 0.50 in Black Redstarts, 0.37 in Common Redstarts and 0.22 in Whinchats. Furthermore, we yielded rarefied lineage richness of 4.66 (± 0.83 SE) for European Robins, 5 (± 0 SE) for Black Redstarts, 5.8 (± 1.06 SE) for Common Redstarts and 6.01 (± 1.25 SE) for Whinchats. The pairwise Chao indices varied between 0.19 and 0.81 (see Fig. 1, right panel).

### Parasitaemia

Individual parasitaemia ranged from 0.001% to 3.2% (median = 0.01%). We found parasitaemia higher than 1% in four of 125 infected hosts—all in Whinchats. Three were infected with the *Haemoproteus* lineage H-ROBIN1, one was infected with lineage P-SW2 of the genus *Plasmodium*.

Parasitaemia of *Haemoproteus* infections was generally higher than *Plasmodium* infections (LM: slope_P→H_ = 0.93 [± 0.19], t = − 4.85, *p* < 0.001). Regardless of parasite genus, parasitaemia in Common Redstarts was lower compared to Whinchats (LM: slope_PhoPho_ = − 0.77 [± 0.20], t = − 3.79, *p* < 0.001). Black Redstarts’ parasitaemia did not significantly differ from Whinchats’ (LM: slope_PhoOch_ = − 0.45 [± 0.26], t = − 1.72, *p* = 0.09). In addition, there was a significant interaction between host species and parasite genus for parasitaemia in European Robins (LM: slope_EriRub_ = − 0.71 [± 0.29], t = − 2.46, *p* = 0.02, slope_Haemoproteus*EriRub_ = 0.87 [± 0.44], z = 1.99, *p* = 0.05), indicating lower parasitaemia in Robins compared to Whinchats for *Plasmodium* infections but not for *Haemoproteus* infections (see Fig. 2, left panel).

The *Plasmodium* lineages P-LK06, P-SaxRub03, P-RTSR1 and P-SGS1 as well as the *Haemoproteus* lineages H-ROBIN1 and H-RS1 caused higher parasitaemia compared to parasitaemia of unknown lineages (LM: slope_LK06_ = 2.06 [± 0.45], t = 4.59, *p* < 0.001; slope_SaxRub03_ = 1.66 [± 0.43], t = 3.88, *p* < 0.001; slope_RTSR1_ = 1.10 [± 0.38], t = 2.89, *p* < 0.01; slope_SGS1_ = 0.42 [± 0.19], t = 2.24, *p* = 0.03; slope_ROBIN1_ = 1.54 [± 0.18], t = 8.40, *p* < 0.001; slope_RS1_ = 0.64 [± 0.31], t = 2.04, *p* = 0.04).

## Discussion

Here, we describe the prevalence and diversity of *Plasmodium* and *Haemoproteus* infections in migratory passerines and provide the first detailed analysis of lineage-specific parasitaemia in these hosts sampled after long endurance flights.

### Factors Correlating with Parasite Prevalence

Besides European Robins, there are few studies with sufficient sample sizes allowing reliable estimation and thus comparison of prevalence for the host species examined in this study. Genus-specific prevalence derived from MalAvi database, suggest that around 8% of European Robins per population or sampled study group were infected with *Haemoproteus* (3.3% in Germany [16], 4.2% in Portugal [17], 9.5% in Slovakia [18] and 13.5% in Sweden [19]) and approximately 6% were infected with *Plasmodium* (1.4% in Portugal [17], 1.6% in Germany [16], 7.7% in Slovakia [18] and 13.5% in Sweden [19]). In a previous study conducted on Ventotene in 1999, Whinchats also had significantly more haemosporidian infections than Common Redstarts (49% in Whinchats, 10% in Common Redstarts; [20]). Therefore, compared to the present study, prevalence was generally lower, but as the earlier study used microscopy alone for detecting parasites, comparing the absolute prevalence values with the current study seems improper. Still, the compilation of literature data suggests that some host species or populations might have consistently higher (e.g., Whinchats) and others consistently lower prevalence (e.g., European Robins), which might at least partly be related to the phylogenetic background of host species [21]. In contrast to predictions from the literature [7, 22], prevalence seemed not to be related to the hosts’ migration strategy (Fig. 1, left panel). Though, a large part of the variation in prevalence might be related to the global zoogeographical region [23] and habitat [24] at the hosts’ breeding and non-breeding residency. Independent of the factors behind, our findings revealed that their remarkably high prevalence and parasite lineage diversity make Whinchats a suitable model species for a multitude of questions related to host–parasite interactions in the African–Palaearctic migration system.

### Factors Related to Lineage Assemblage

We found most of the lineages to be unique to one host species, few were shared between two hosts and two lineages were ubiquitously shared among all four host species. Each of the four new lineages was unique to only one of the host species (i.e., Common Redstarts and Whinchats). With 99% matching base pairs, the lineage P-PhoPho01 is most similar to a common *Plasmodium* lineage P-SW2, found in all four studied host species (see further below). The lineage P-SaxRub01 is most similar to P-GRW07 (98% of base pairs matching), according to MalAvi, a lineage previously found in migratory species of the families Sylviidae and Muscicapidae in Europe. Finally, P-SaxRub02 and P-SaxRub03 are both matching by 99% with the lineage P-SYBOR05, a widespread *Plasmodium* lineage previously detected in migratory Garden Warblers (*Sylvia borin*) in Europe and Africa [25], as well as migratory Muscicapidae species in Europe [26] and a resident Timaliidae species in South Africa [27].

The two lineages shared among all four host species in our study, are the generalist *Plasmodium* lineages P-SGS1 and P-SW2. According to the MalAvi database, the lineage P-SGS1 is one of the most widespread haemosporidian lineage found occurring in eleven avian orders (Passeriformes, Gruiformes, Galliformes, Sphenisciformes, Procellariiformes, Ciconiiformes, Anseriformes, Strigiformes, Trochiliformes, Charadriiformes and Columbiformes) and six continents (Asia, Europe, Oceania, Africa, South and North America). With seven orders (Passeriformes, Strigiformes, Gruiformes, Pelecaniformes, Ciconiiformes, Anseriformes and Charadriiformes) and three continents (Europe, Africa and Asia) P-SW1 is less pervasive, but not surprisingly shared among four Muscicapidae host species, all from the Afro-Palaearctic migration system.

The little sharing of lineages among few host species in our study is likely related to the fact that these birds have different breeding origins and destinations, each with different vector pools. The birds in our study were randomly sampled on a passage site, where they merely stopover for very short time [28], wherefore local transmission might be negligible. This contrasts with community studies conducted at breeding or non-breeding residencies would typically show a higher degree of lineage sharing for closely related host species [19].

There was no obvious similarity in the lineage assemblage among more closely related hosts. Furthermore, we did not found higher similarity in the lineage assemblages or in lineage diversity among hosts with the same migration strategy in our study. Potentially, the differences in habitat types and vector pools faced by short- and long-distance migrants might not be so pronounced as they have previously been found between resident hosts and obligate migrants [29, 30].

### Factors Correlating with Parasitaemia

Parasitaemia significantly differed between the parasite genera and genetic lineages but also between certain host species. Average parasitaemia was considerably higher for *Haemoproteus* as compared to *Plasmodium* infections, a pattern well-established by numerous previous studies [31–34]. Even though this pattern seems rather consistent across diverse host species, the causative factors remain largely unknown and might be found mainly in parasite biology. A prominent life-cycle difference between the two parasite genera*,* which could cause this variation in chronic parasitaemia levels, are the means of asexual proliferation: while merogony of *Haemoproteus* takes place in tissue cells of the hosts’ inner organs, *Plasmodium* asexually proliferates in erythrocytes [35]. This could entail varying costs of hosting gametocytes in the peripheral blood (potentially lower costs for *Haemoproteus*) and/or the longevity of circulating gametocytes (potentially longer for *Haemoproteus*; as hypothesised by [36, 37]). Both aspects could finally lead to the observed genus-specific chronic parasitaemia. However, both longevity of parasite cells and parasite costs in specific host compartments are difficult to study, and thus the genus-related patterns in chronic parasitaemia levels still remain to be unravelled.

In addition to the genus-specific parasitaemia levels, we found distinct lineage-specific parasitaemia ranges. For instance, the parasitaemia of H-RS1 varied between 0.002% and 0.25% (median = 0.02%), while H-ROBIN1 ranged between 0.003% and 3.2% (median = 0.29%). Therefore, the maximum parasitaemia of one lineage barely reaches the median of the other, even though they both belong to the same genus. On the other hand, we found lineages like P-LK06 to range from 0.03% to 0.97% (median = 0.74%) and P-SW2 from 0.001% to 1.14% (median = 0.003). While maximal parasitaemia was very similar, the medians varied by several orders of magnitude. As most lineages were uniquely infecting a single host species (Fig. 1, right panel), we assume that lineage-specific parasitaemia reflects a combination of lineage pathogenicity [38, 39] and host immunocompetence ([40] and references therein).

The host species-related differences in parasitaemia we found here could just reflect the varying relative abundances of lineages they host. For instance, the relatively low parasitaemia in Common Redstarts is rather a result of being a frequent host to relatively low-pathogenic lineages (like, e.g., H-RS1) and not lineages causing higher parasitaemia (like H-ROBIN1). However, a dataset with more shared lineages of very variable parasitaemia ranges would be needed to substantiate such differences to be caused by high host species-specific immunity and ability to suppress parasitaemia of otherwise high-pathogenic lineages.

Interestingly, we found some individuals with high parasitaemia, exceeding levels typical for chronic haemosporidian infections, which were obviously able to cross major ecological barriers. This is unexpected as we captured birds with stationary mist nets, which is only suitable for active birds. Mist-netting is often described to induce sampling bias, yielding only birds with low parasitaemia infections not impairing their mobility [35]. Yet, as most parasitaemia data come from infection experiments and data from natural infections remain rare, it is difficult to set a threshold for delimiting chronic from acute infections. According to the literature, chronic parasitaemia levels of *Haemoproteus* and *Plasmodium* infections in wild birds are not above 1–2%, usually less than 0.1% [33, 35]. Three of the four individuals with parasitaemia values above 1% had *Haemoproteus* infections (lineage H-ROBIN1), for which this might be still in the range of chronic infections. For *Plasmodium* infections such high parasitaemia is often only reached in the course of an acute infection [41, 42], either acquired just before departure from the non-breeding site or relapsing from a chronic infection. In our study, the lineages P-LK06 reached high parasitaemia relatively often, with two out of three infections we found above 0.5% parasitaemia. The second lineage P-SW2 caused high parasitaemia more rarely, with two out of 13 infections in our study reaching parasitaemia above 0.5%.

The lineage P-LK06 is particularly common in the Mediterranean region, in resident birds in Morocco [17] and both from insular and continental Spain and Portugal [43, 44]. Therefore, it seems clear that transmission takes place in this area. The only record from more northern latitudes comes from two adult long-distance migratory Common Whitethroats (*Sylvia communis*) in southern Sweden [19], so interference about transmission is not possible. Even though the lineage P-SW2 is much more widespread than P-LK06, the only transmission area that can be deduced from MalAvi records is southern Sweden [19, 45, 46]. Taken this knowledge about potential transmission areas into account, the high-parasitaemia records in our study are very probable relapses of infections previously acquired during the breeding period rather than recent primary infections transmitted during non-breeding. This also fits to the idea that the spring migration period with its physiologically challenging endurance flights [47] and seasonal changes in hormones in preparation for breeding [48] is a period of reduced immune function in birds and may be associated with infection relapses.

In conclusion, our study added a substantial amount of basic data about lineage-specific parasitaemia of natural infections in migratory songbirds. The combined application of molecular detection and classic microscopy for quantifying parasite infections in peripheral blood stream is still worthwhile. Future studies using such a complementary approach can hopefully result in new insights into factors and conditions affecting avian haemosporidian parasitaemia for a broader spectrum of host–parasite associations in the wild.

## Data Availability

The datasets generated and analysed in this study are deposited and publicly available from the MalAvi database with reference to this publication.
